# Editorial: Genetic features contributing to eye development and disease

**DOI:** 10.3389/fcell.2022.1008907

**Published:** 2022-09-09

**Authors:** Wenchang Xu, Xinqi Liu, Wenjuan Han, Ling Zhao

**Affiliations:** ^1^ State Key Laboratory of Ophthalmology, Zhongshan Ophthalmic Center, Sun Yat-sen University, Guangdong Provincial Key Laboratory of Ophthalmology and Visual Science, Guangzhou, China; ^2^ Guangdong Province Key Laboratory of Brain Function and Disease, Zhongshan School of Medicine, Sun Yat-sen University, Guangzhou, China

**Keywords:** eye diseases, eye development, causal genes, susceptibility genes, genetic mechanisms

Growing evidence has shown that genetic factors play crucial roles in the disorder of eye development and the progression of ocular diseases (Singh and Tyagi, 2018). Ocular disorders with complex inheritance are responsible for most blindness, however, there are currently no cures for many of these conditions (Singh and Tyagi, 2018; Chen et al., 2021). A better understanding of the genetic underpinnings of ocular diseases could facilitate the accurate diagnosis, counseling and treatment of these diseases.

The eye consists of three main types of tissues: 1) refracting tissues that focus incoming light onto light-sensitive tissues (including the pupil, iris, lens, ciliary muscle, cornea, vitreous and aqueous fluid), 2) light-sensitive tissues that convert detected light into electrical signals and transmit them to the brain (including the retina and optic nerve), and 3) support tissues that provide the architectural support for the shape of the eyeball (including the sclera, conjunctiva and uvea) (Rocher, 2010). These parts in the eyes must work together to produce a clear vision.

This topic summarizes ten original research articles that explored the genetic effects and mechanisms of genetic factors contributing to eye development and disease from diverse aspects, providing new insights into treating eye diseases (Table 1). According to the research object, these studies can be divided into three categories: novel causal and susceptibility genes in eye diseases, genetically engineered animal models for eye diseases, and novel concepts or innovative approaches for eye development and diseases.

**TABLE 1:** Genes studied in the article collection.

Gene name	Eye development or diseases	Article
*OPA1*	Ethambutol-induced optic neuropathy	Wei et al.
*BMP4*	Syndromic microphthalmia and pathologic myopia	Zhang et al.
*GJA8*	Congenital hereditary cataract	Jin et al.
*FBN1*	Congenital ectopia lentis	Jiang et al.
*MDM2*	Epimacular membranes	Luo et al.
*LSS*	Congenital hereditary cataract	Zhao et al.
*KCNJ13*	Snowflake Vitreoretinal Degeneration and Leber congenital amaurosis	Hejtmancik et al.
*ASRGL1*	Retinitis pigmentosa	Zhu et al.
*Atoh7 and Brn3b*	*In vivo* regeneration of functional RGCs	Xiang et al.
Transcriptome atlas of the hRPE	Human retinal disease	Hou et al.

## Novel causal and susceptibility genes in eye diseases

Inherited eye diseases affect approximately one in 1,000 people worldwide, but the molecular mechanisms underlying most of them remain unclear (Mejecase et al., 2020). Identifying novel causal and susceptibility genes allows a better understanding of the disorders and offers new clues for better-targeted disease management.

Wei et al. identified mitochondrial mutations (*OPA1* and LHON-mtDNA) in nearly half of patients with ethambutol-induced optic neuropathy (EON), a well-recognized ocular complication associated with ethambutol treatment in tuberculosis patients. Since some patients with EON have severe and permanent visual loss even without the known risk factors, their findings that mitochondrial genetic variations are major predisposing factors for the development of EON provided a better understanding of EON and additional support for genetic counseling.

Zhang et al. found variants in *BMP4* contribute to a novel phenotype of pathologic myopia rather than syndromic microphthalmia that have been reported in a previous study (Reis et al., 2011). The observations that mutations in the same gene could cause both syndromic microphthalmia and pathologic myopia suggested bidirectional roles of *BMP4* in early ocular development and provided new insight into the disease mechanism.

Jin et al. identified a novel connexin 50 mutation P88L in patients with congenital cataract and analyzed the function of this mutation. Congenital hereditary cataract is a heterogeneous disorder and the most common cause of childhood blindness (Berry et al., 2020). Their findings expand the spectrum of pathogenic connexin 50 mutations in congenital cataract and provide additional support for clinical diagnosis and genetic counseling.

Congenital ectopia lentis (CEL), the second leading cause of pediatric lens surgery after congenital cataracts, could be caused by mutations in cbEGF-like domains of fibrillin-1 (*FBN1*) (Faivre et al., 2008). However, the correlations between genotype and phenotype of cbEGF-like mutations remain unknown. Jiang et al. focused on clinical manifestations of CEL in patients with different mutations in cbEGF-like domains of *FBN1*. And they clarified the correlations between genotype and phenotype for cbEGF-like mutations. Their work increases our knowledge of CEL and offers new clues for the targeted treatment of the disease.

Luo et al. presented work on the role of the *mouse double minute 2* (*MDM2*) gene single nucleotide polymorphism (SNP) T309G in the development of epimacular membranes (EMMs), relatively common sight-threatening conditions characterized by fibrocellular proliferation along the surface of the internal limiting membrane (ILM) of the retina. They first reported that the *MDM2* SNP309 G allele was a risk allele for EMM in a Chinese population. Their observation provides new insights into the molecular mechanism underlying these pathologies.

## Genetically engineered animal models for eye diseases

Since the experimental studies of many inherited eye disorders in human beings are limited, the availability of genetically engineered animal models is very valuable for exploring the pathogenic mechanisms of these conditions and developing translational therapies.

Zhao et al. generated a knock-in mouse model with lanosterol synthase (Lss) G589S mutation, which can recapitulate human congenital cataract resulting from G588S mutation in human LSS. Mice with homozygous Lss G589S mutation exhibited disrupted lens fiber differentiation at early-stage of lens development and down-regulated cholesterol synthesis signaling pathways. Their findings elucidate the important roles of LSS in lens development, contributing to a better understanding of LSS defects and disturbed sterol signaling in cataractogenesis and the development of therapies for cataracts.

Mutations in KCNJ13 are responsible for both snowflake vitreoretinal degeneration (SVD) and Leber congenital amaurosis (LCA) (Hejtmancik et al., 2008; Sergouniotis et al., 2011). Existing animal models have not been able to well verify causality and dissect the mechanisms and pathogenesis of these diseases. Hejtmancik et al. generated and characterized the Kcnj13 knockout mouse and RPE-specific conditional Kcnj13 knockout mice. Their work provides a potential mouse model system for elucidating the pathology of these diseases and developing gene therapy trials.

G178R in asparaginase and isoaspartyl peptidase 1 (*ASRGL1*) has been reported as a causing mutation for retinitis pigmentosa (RP), an inherited retinal degenerative disease for which there is currently no cure (Biswas et al., 2016). Since the pathological and molecular mechanisms of *ASRGL1* in causing RP remains unknown, Zhu et al. developed *Asrgl1* knockout mice and explored the function of *Asrgl1* in the mammalian retina. Their findings provide a knockout mouse model for improving the understanding of RP disease mechanisms.

## Novel concepts or innovative approaches for eye development and diseases

Novel concepts or innovative approaches could provide new insights into understanding the mechanism of eye development and diseases, and developing new diagnostic and treatment strategies.

Glaucoma is the most common cause of irreversible blindness worldwide and irreversible degeneration of retinal ganglion cells (RGCs) and the optic nerve are the common features shared by all forms of glaucoma (Jonas et al., 2017). *In vivo* RGC regeneration would be an ideal therapy but mammalian retinas are thought to lack regenerative capacity. Xiang et al. demonstrated that endogenous mouse Müller glia (MG) could be reprogramed into functional retinal ganglion cells (RGCs) *in vivo* by Math5 and Brn3b together, two crucial transcription factors (TFs) involved in retinal ganglion cell (RGC) generation and differentiation (Yang et al., 2003; Pan et al., 2008). Their study provides a promising new therapeutic approach for visual restoration in patients with glaucoma and other optic neuropathies.

Hou et al. profiled a single-cell transcriptome atlas of human RPE (hRPE) cells and provided a map of disease-related genes in the hRPE. They found two subpopulations, the macular RPE and peripheral RPE clusters which exhibited substantial differences in gene expression patterns and functions, play crucial roles in the potential treatment of retinal diseases. Their work provides important information for understanding the cellular mechanisms and treating pathological conditions of the hRPE associated with ocular diseases.

## Conclusion

These research articles on the topic show that elucidating the genetic underpinnings of ocular disorders leads to a better understanding of these diseases, which will contribute to clinical diagnosis, genetic counseling, early intervention and targeted treatment. The application and advancement of integrated multi-omics will further improve our knowledge of complex traits and provide new insights into the pathogenesis of ocular diseases. New genetic methodologies based on automated methods are expected to be developed for accurate and routine diagnosis of eye diseases that have highly diverse genetic causes and are difficult to identify. And gene and cell therapies will open new doors for the treatment of currently incurable eye disorders.
